# Supplementary material for: Ganoderic Acid Ameliorates Ulcerative Colitis by Improving Intestinal Barrier Function via Gut Microbiota Modulation
Source: Int J Mol Sci. 2025 Mar 10;26(6):2466. doi: 10.3390/ijms26062466 (PMC11942431; doi:10.3390/ijms26062466)
Supplement: Supplementary file 1 [file ijms-26-02466-s001.zip › ijms-3438793-supplementary.pdf]

International Journal of
Molecular Sciences

Supplementary Figure

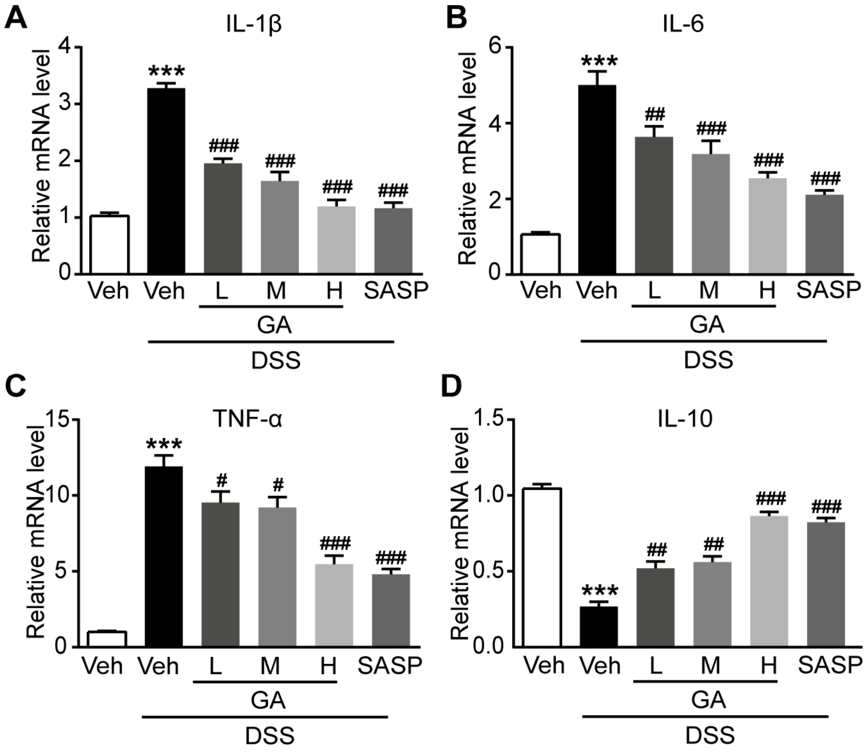

Supplementary Figure S1. Effect of GA on colonic inflammation in UC mice. (A) Relative mRNA level of IL-1β. (B) Relative mRNA level of IL-6. (C) Relative mRNA level of TNF-*α*. (D) Relative mRNA level of IL-10. Values are shown as the mean ± SEM (n = 12). ***P < 0.001 vs Veh group. #P < 0.05, ##P < 0.01, ###P < 0.001 vs DSSVeh group.

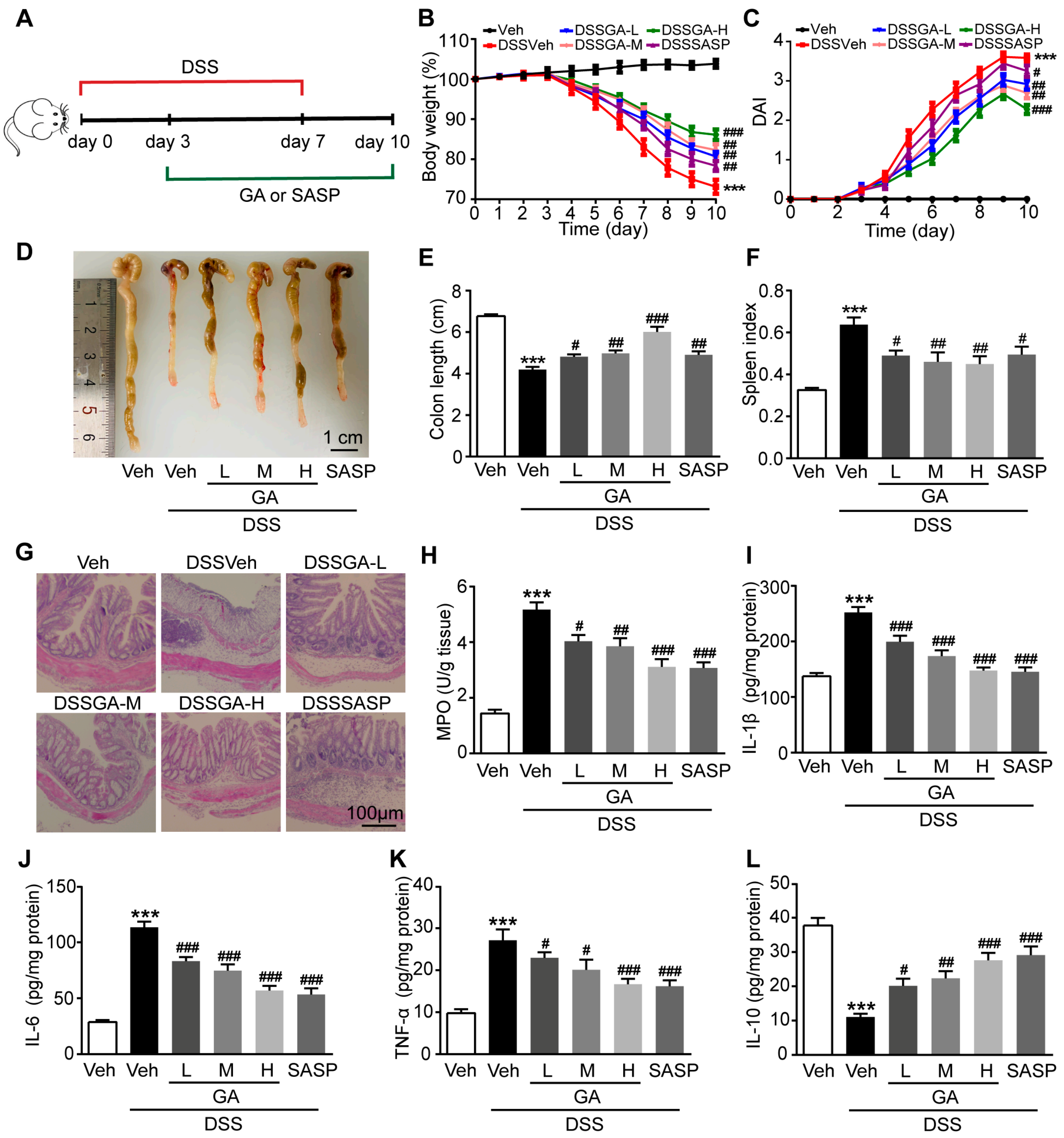

Supplementary Figure S2. The therapeutic effect of GA on UC mice. (A) Experimental procedure for studying the effect of GA on DSS-induced UC in mice. (B) Daily changes in body weight. (C) DAI. (D) Colon appearance, Scale bar = 1 cm. (E) Colon length. (F) Spleen index. (G) Representative pictures of H&E-stained colon tissue (magnification of ×100). Scale bar = 100 μm. (H) MPO activity. (I) IL-1β level. (J) IL-6 level. (K) TNF-*α* level. (L) IL-10 level. Values are shown as the mean ± SEM (n = 12). ***P < 0.001 vs Veh group. #P < 0.05, ##P < 0.01, ###P < 0.001 vs DSSVeh group.

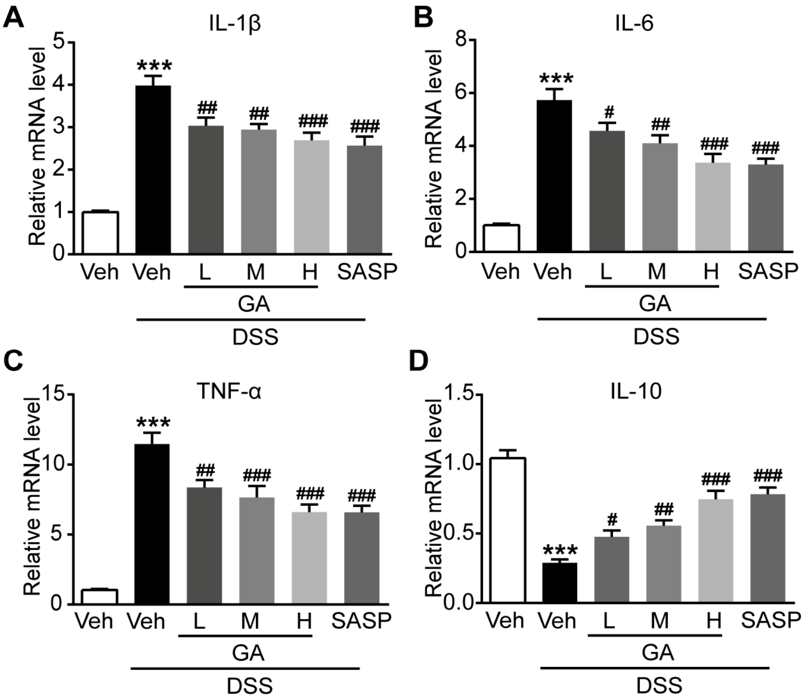

Supplementary Figure S3. Effect of GA on mRNA transcript levels of colonic inflammatory cytokines in therapeutic experiments. (A) Relative mRNA level of IL-1β. (B) Relative mRNA level of IL-6. (C) Relative mRNA level of TNF-α. (D) Relative mRNA level of IL-10. Values are shown as the mean ± SEM (n = 12). ***P < 0.001 vs Veh group. ##P < 0.01, ###P < 0.001 vs DSSVeh group.

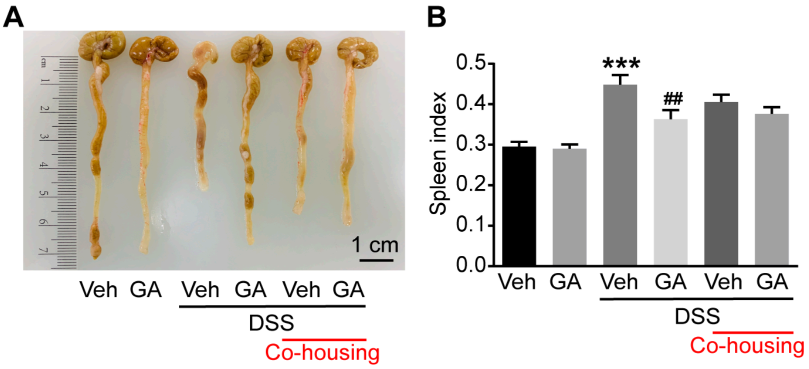

Supplementary Figure S4. Effect of co-housing on anti-UC activity of GA. (A) Colon appearance. Scale bar = 1 cm. (B) Spleen index. Values are shown as the mean ± SEM (n = 12). ***P < 0.001 vs Veh group. ##P < 0.01 vs DSSVeh group.

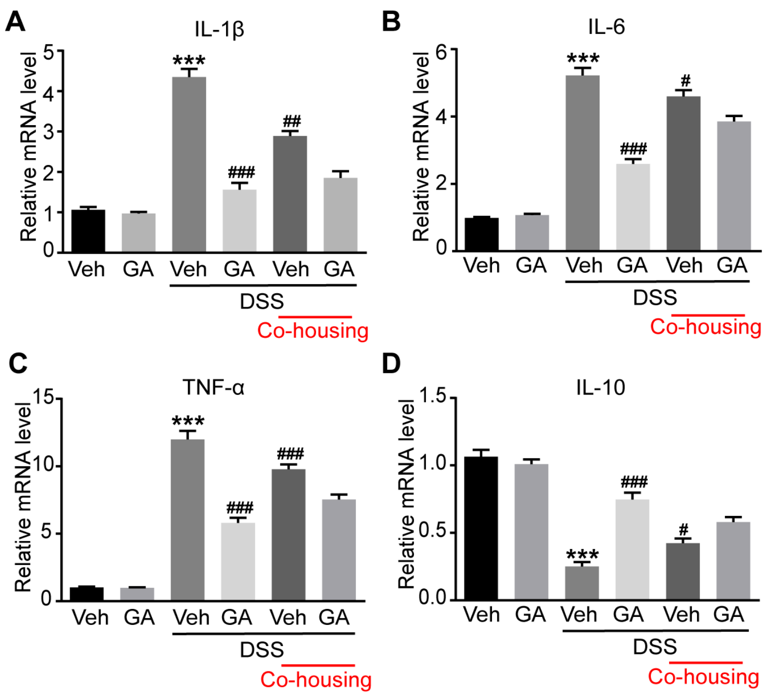

Supplementary Figure S5. Effect of co-housing on mRNA transcript levels of colonic inflammatory cytokines. (A) Relative mRNA level of IL-1β. (B) Relative mRNA level of IL-6. (C) Relative mRNA level of TNF-α. (D) Relative mRNA level of IL-10. Values are shown as the mean ± SEM (n = 12). ***P < 0.001 vs Veh group. ##P < 0.01, ###P < 0.001 vs DSSVeh group.

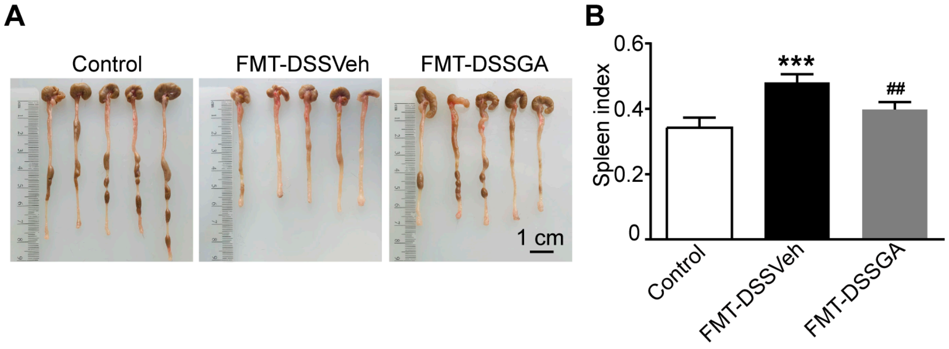

Supplementary Figure S6. Effect of FMT from the GA-treated donor mice on pathological symptoms of UC mice. (A) Colon appearance. Scale bar = 1 cm. (B) Spleen index. Values are shown as the mean ± SEM (n = 12). ***P < 0.001 vs Control group. ##P < 0.01 vs FMT-DSSVeh group.

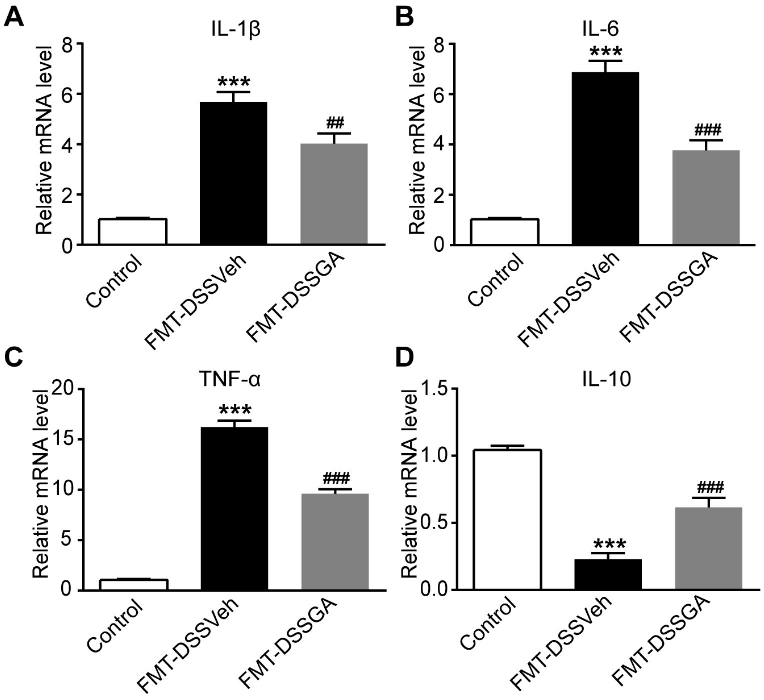

Supplementary Figure S7. Effect of FMT from the GA-treated donor mice on mRNA transcript levels of colonic inflammatory cytokines in therapeutic experiments. (A) Relative mRNA level of IL-1β. (B) Relative mRNA level of IL-6. (C) Relative mRNA level of TNF-α. (D) Relative mRNA level of IL-10. Values are shown as the mean ± SEM (n = 12). ***P < 0.001 vs the Control group. ##P < 0.01, ###P < 0.001 vs FMT-DSSVeh group.
